# Homoplastic microinversions and the avian tree of life

**DOI:** 10.1186/1471-2148-11-141

**Published:** 2011-05-25

**Authors:** Edward L Braun, Rebecca T Kimball, Kin-Lan Han, Naomi R Iuhasz-Velez, Amber J Bonilla, Jena L Chojnowski, Jordan V Smith, Rauri CK Bowie, Michael J Braun, Shannon J Hackett, John Harshman, Christopher J Huddleston, Ben D Marks, Kathleen J Miglia, William S Moore, Sushma Reddy, Frederick H Sheldon, Christopher C Witt, Tamaki Yuri

**Affiliations:** 1 Department of Biology, University of Florida, Gainesville, FL 32611, USA; 2 Department of Mathematics, University of Florida, Gainesville, FL 32611, USA; 3 Zoology Department, Field Museum of Natural History, 1400 S. Lakeshore Drive, Chicago, IL 60605, USA; 4 Museum of Vertebrate Zoology and Department of Integrative Biology, University of California, Berkeley, Berkeley, CA 94720, USA; 5 Department of Vertebrate Zoology, Smithsonian Institution, 4210 Silver Hill Road, Suitland, MD 20746, USA; 6 Behavior, Ecology, Evolution, and Systematics Program, University of Maryland, College Park, MD 20742, USA; 7 4869 Pepperwood Way, San Jose, CA 95124, USA; 8 Museum of Natural Science and Department of Biological Sciences, 119 Foster Hall, Louisiana State University, Baton Rouge, LA 70803, USA; 9 Department of Biological Sciences, Wayne State University, 5047 Gullen Mall, Detroit, MI 48202, USA; 10 Biology Department, Loyola University Chicago, Chicago, IL 60626, USA; 11 Department of Biology and Museum of Southwestern Biology, University of New Mexico, Albuquerque, NM 87131, USA; 12 Sam Noble Oklahoma Museum of Natural History, University of Oklahoma, Norman, OK 73072, USA

## Abstract

**Background:**

Microinversions are cytologically undetectable inversions of DNA sequences that accumulate slowly in genomes. Like many other rare genomic changes (RGCs), microinversions are thought to be virtually homoplasy-free evolutionary characters, suggesting that they may be very useful for difficult phylogenetic problems such as the avian tree of life. However, few detailed surveys of these genomic rearrangements have been conducted, making it difficult to assess this hypothesis or understand the impact of microinversions upon genome evolution.

**Results:**

We surveyed non-coding sequence data from a recent avian phylogenetic study and found substantially more microinversions than expected based upon prior information about vertebrate inversion rates, although this is likely due to underestimation of these rates in previous studies. Most microinversions were lineage-specific or united well-accepted groups. However, some homoplastic microinversions were evident among the informative characters. Hemiplasy, which reflects differences between gene trees and the species tree, did not explain the observed homoplasy. Two specific loci were microinversion hotspots, with high numbers of inversions that included both the homoplastic as well as some overlapping microinversions. Neither stem-loop structures nor detectable sequence motifs were associated with microinversions in the hotspots.

**Conclusions:**

Microinversions can provide valuable phylogenetic information, although power analysis indicates that large amounts of sequence data will be necessary to identify enough inversions (and similar RGCs) to resolve short branches in the tree of life. Moreover, microinversions are not perfect characters and should be interpreted with caution, just as with any other character type. Independent of their use for phylogenetic analyses, microinversions are important because they have the potential to complicate alignment of non-coding sequences. Despite their low rate of accumulation, they have clearly contributed to genome evolution, suggesting that active identification of microinversions will prove useful in future phylogenomic studies.

## Background

**Figure 1:**
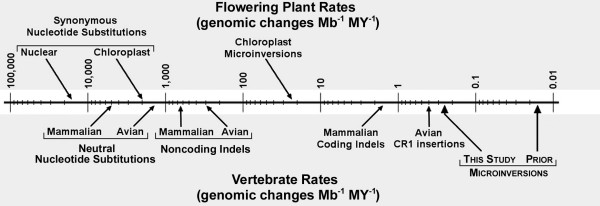
**Approximate rates of accumulation for different genomic changes over evolutionary time**. Details of the literature survey used to estimate these rates are provided in Additional file 2. The estimate of the avian microinversion rate reflects the results of this paper. Estimates of evolutionary rates for nucleotide substitutions and indels in birds appear lower than those for mammals, consistent with some previous publications [59], but it is important to note that substantial rate variation occurs within each group (e.g., [27,60]). As described in the text, it may be better to interpret prior estimates of the mammalian microinversion rate as the rate at which relatively long microinversions accumulate.

Reconstructing the evolutionary relationships among organisms and changes in their genomes are major goals of phylogenomics [1-3]. The characteristics of genomes that have been used to reconstruct evolutionary history reflect the multitude of changes that arise due to distinct mutational mechanisms and accumulate at a variety of rates (Figure 1). The most slowly accumulating changes, collectively designated rare genomic changes (RGCs), reflect a heterogeneous set of mutational processes. RGCs include transposable element insertions (e.g., Kriegs et al. [4]), gene order changes [5], and additional less-studied phenomena [6-8]. Microinversions [6] are one of these relatively poorly-studied types of RGCs.

Despite this heterogeneity, RGCs are thought to exhibit less homoplasy (evolutionary convergence and reversals) than nucleotide substitutions [9]. Indeed, some RGCs have been viewed as "perfect" homoplasy-free (or virtually homoplasy-free) characters. Establishing that specific types of RGCs, like microinversions, are perfect characters is important for two reasons. First, it would provide information about the mutational and evolutionary processes that underlie their accumulation, illuminating processes that contribute to genome evolution. Second, perfect RGCs could provide a practical means to assemble the tree of life because phylogenetic reconstruction is straightforward when homoplasy is absent [6].

Even perfect RGCs can appear homoplastic when found in genomic regions with an evolutionary history incongruent with the species tree [5,10]. The appearance of homoplasy due to incomplete lineage sorting, called hemiplasy [11], typically occurs in trees with short internal branches [12,13]. However, rapid radiations with short internal branches ("bushes" or "biological big bangs") may be relatively common events in the tree of life [14,15]. This suggests that analyses of RGC data should consider hemiplasy explicitly.

Microinversions are defined as cytologically undetectable inversions [6], although in practice the size range considered depends on the type of data examined and method used for detection. Feuk et al. [16] classified inversions ranging in size from 23 base pairs (bp) to 62 megabases (Mb) as microinversions, whereas Ma et al. [1] considered all inversions greater than 50 kilobases (kb) to be "large" inversions rather than microinversions. The lower limit also varies, going down to 4 bp [17]. Not surprisingly, studies using whole genomes (e.g., [1,16]) have identified larger inversions, while phylogenetic studies (often restricted to a single locus or region of an organellar genome) have typically revealed much smaller microinversions (e.g., [17-21]). Nonetheless, the size spectra reported for genome-scale and phylogenetic studies overlap, suggesting that both types of studies include at least some inversions that result from similar biological phenomena. Using the term "microinversion" to refer to inversions that are long enough to include one or more complete genes seems inappropriate, suggesting that it should be reserved for shorter inversions. However, this criterion may be difficult to apply in practice, since the length of genes exhibits substantial variation among organisms and within genomes. The majority of genes are <50 kb in length in most vertebrate lineages, suggesting that the Ma et al. [1] size criterion may be appropriate and simple to use. Therefore, we recommend using 50 kb as the maximum size for microinversions in most vertebrate genomes, although we also note that the most appropriate size criterion is likely to depend upon the focal organism.

The hypothesis that microinversions and other RGCs are perfect characters reflects both their large state space (number of potential character states) and slow rate of accumulation over evolutionary time, making independent changes to the same state unlikely. The state space for different RGCs will depend upon the details of each type of genomic change, but it seems likely that the state space for microinversions is large; they can be of a variety of lengths and have any specific nucleotide for endpoints, making it unlikely that independent microinversions will appear identical. Previous studies have also suggested that microinversions accumulate at a very low rate (Figure 1), although this observation may be biased by the size spectrum of the inversions that were identified and considered to be microinversions. Ma et al. [1] reported that smaller microinversions (they identified inversions as short as 31 bp) occur more frequently than larger ones. However, the rate of accumulation for inversions that are even shorter than those identified by Ma et al. [1] remains unclear and these differences among previous studies make direct comparisons challenging. Nonetheless, it seems certain that microinversions accumulate at least several orders of magnitude more slowly than nucleotide substitutions. Thus, the hypothesis that microinversions are perfect characters that will be very useful for assembling the tree of life remains reasonable.

The mechanism(s) responsible for microinversion accumulation remain poorly characterized, making empirical tests of the "perfect character hypothesis" for these relatively poorly studied RGCs critical. Indeed, homoplastic microinversions have been identified in angiosperm chloroplast genomes [17,19], in contrast to expectation based upon the perfect character hypothesis. Most chloroplast microinversions appear to be associated with palindromic sequences that have the potential to form stem-loop structures in transcripts [17,19] and these palindromes may facilitate inversion. Indeed, Catalano et al. [21] reported that microinversions are correlated with higher stability of the hairpins that have the potential to form at these stem-loop regions, in agreement with the hypothesis that hairpin formation facilitates inversion. Since many chloroplast stem-loop structures have regulatory functions (e.g., Stern et al. [22]) they are typically conserved, creating the potential for recurrent inversions at specific sites. Regulatory stem-loops are present in vertebrate introns (e.g., Hugo et al. [23]) and at least one vertebrate microinversion noted in a vertebrate phylogenetic study was associated with an inverted repeat [18]. However, conserved stem-loops appear to be uncommon in vertebrate introns whereas chloroplast stem-loops are relatively common [22,24]. This difference is consistent with the observation that few animal microinversions appear homoplastic [6,25]. Indeed, all microinversions observed in those studies were either homoplasy-free or conflicted with short branches. Thus, the small number of animal microinversions that appear to conflict with the species tree based upon other data may result from hemiplasy rather than homoplasy. Thus, microinversions in animal nuclear genomes remain candidates for "ideal RGCs", able to recover branches in gene trees accurately.

Microinversions can be difficult to identify, making the study of these interesting and phylogenetically useful genomic changes challenging. In fact, ~80% of the inversions identified in the Feuk et al. [16] comparison of the human and chimpanzee genomes were later suggested to be contig assembly artifacts [6]. This problem can be solved by restricting the term microinversion to the shortest part of the inversion spectrum, limiting the maximum size of the microinversions to less than the length of an individual sequencing read (i.e., focusing on inversions that are <400 bp for Sanger sequencing). Comparing closely related taxa also has the potential to facilitate microinversion identification. Indeed, most microinversions identified in a comparison of four mammalian genomes were found in the two most closely related taxa [1]. Here we use these strategies to identify microinversions in non-coding regions associated with 17 loci from 169 birds. We examined variation among loci in the microinversion rate (hereafter abbreviated λ_MI_), identified phylogenetically informative and homoplastic microinversions, and found evidence that the number of microinversions has been underestimated in previous large-scale studies.

## Methods

### Sequencing, Alignment and Microinversion Identification

We primarily used published data [26-28], although some novel *CLTCL1 *sequences were generated using the primers and PCR conditions from Kimball et al. [29] (for details, see Additional file 1). For this study, we focused on shorter sequences with extensive taxon sampling (Table 1) instead of complete genomic sequences [26-28]. Sequences were aligned manually, sometimes starting from an alignment produced in an automated manner (i.e., using Clustal [30] or MAFFT [31]). Alignments were refined iteratively with input from at least two different individuals. During this process alignments were examined carefully; this resulted in the identification of a number of microinversions "by eye" (Additional file 2, Table S2).

**Table 1:** Estimates of the microinversion rate (λ_MI_) for different loci.

Locus	Chr^*a*^	Mean Non-coding Length (bp)	Treelength (MY)^*b*^	# of Inversions^*c*^	Estimated Rate (λ_MI_) (inversions Mb^-1 ^MY^-1^)
*CLTCL1*	15	360	8890	5	1.58
*CLTC*	19	1310	9280	19	1.56
*PCBD1*	6	800	9150	5	0.68
*HMGN2*	23	1340	5400	4	0.55
*EEF2*	28	1210	9230	6	0.54
*IRF2*	4	600	9090	2	0.37
*GH1*	27	1030	9090	3	0.32
*ALDOB*	Z	1450	8850	4	0.31
*TPM1*	10	450	8090	1	0.28
*FGB*	4	2070	9360	4	0.21
*TGFB2*	3	560	9360	1	0.19
*CRYAA*	1	930	8740	0	0
*EGR1*	13	490^*d*^	8970	0	0
*MB*	1	680	9190	0	0
*MUSK*	Z	510	8810	0	0
*MYC*	2	620^*d*^	9240	0	0
*RHO*	12	1190	8990	0	0

Overall	--	15600	--	54	0.39
Excluding hotspots^*e*^		13930	--	30	0.25

^*a *^Chromosomal location in the chicken (*Gallus gallus*).

^*b *^Sum of the branch lengths after rate smoothing in millions of years (MY). Divergence times were calibrated by assuming of a mid-Cretaceous (~100 MYA) origin of Neoaves. Differences among loci reflect the amounts of missing data.

^*c *^The number of inversion events based upon the MP criterion.

^*d *^The non-coding portions of two loci (*EGR1 *and *MYC*) include 820 bp of 3' UTR. All *EGR1 *non-coding sequence is 3' UTR and about half (330 bp) of *MYC *non-coding sequence is 3' UTR.

^*e *^*CLTC *and *CLTCL1 *were excluded for this estimate.

**Figure 2:**
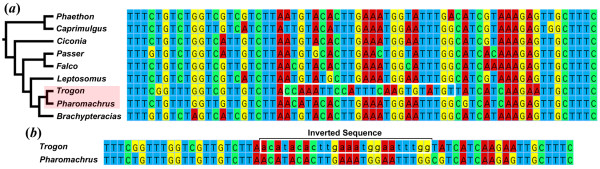
**Example of a microinversion**. (a) A conserved region in *TPM1 *intron 6 with a 24 bp microinversion (outlined in white) in *Trogon personatus*. (*b*) Inverting the *Trogon *sequence (indicated in lower-case) results in a sequence identical to *Pharomachrus auriceps*, its sister taxon in the tree.

Microinversions were also identified by a computational method that combined the multiple sequence alignments with the results of complementary strand alignments for all pairs of sequences (Additional file 2, Figure S1). The pairwise complementary strand alignments were generated using bl2seq [32] and YASS [33] and mapped onto the multiple sequence alignments using a program written by ELB. This program saved a table that included the first and last positions of each pairwise complementary strand alignment in the multiple sequence alignment and highlighted the overlapping pairwise complementary strand alignments (an example is presented in Additional File 3 along with a description of the algorithm in pseudocode). Microinversions are expected to result in complementary strand alignments that either overlap or are located near each other in the sequence alignment. The presence or absence of microinversions at each position identified as a significant complementary strand hit involving sequences that were overlapping or located near each other in the multiple sequence alignment was then validated by visual inspection. Microinversion endpoints were assigned based upon the length of the complementary strand alignments, although there were some cases where inversion endpoints were difficult to identify (e.g., Figure 2). Validating microinversions shorter than 5 bp was difficult, so that was the minimum size considered.

The DNA mfold server (http://mfold.bioinfo.rpi.edu/cgi-bin/dna-form1.cgi; [34]) was used to search for stem-loop structures, and the MEME server (http://meme.sdsc.edu/meme4_4_0/intro.html) was used to search for sequence motifs that might be associated with inversions.

### Patterns and Rates of Microinversion Evolution

Microinversions were coded as binary characters, and PAUP* 4.0b10 [35] was used to calculate numbers of inversion events using maximum-parsimony (MP) and the Hackett et al. [27] topology. λ_MI _was expressed as microinversions Mb^-1 ^MY^-1 ^to facilitate comparison to other studies [6]. The null hypothesis of equal genome-wide microinversion rates was tested as described by Han et al. [36]. Briefly, a global Poisson model (which assumes equal genome-wide rates) was used as the null hypothesis, and the fit of that null model was compared to that of the more general negative binomial (NB) model (which permits variation in λ_MI_) using a likelihood ratio test (LRT). See Additional file 2 for details.

### Phylogenetic Analyses

Phylogenetic analyses of the *CLTC *alignment, conducted to provide an estimate of the *CLTC *gene tree, used RAxML 7.0.4 [37]. Microinversions and sites with gaps and/or missing data in more than 50% of taxa were excluded before conducting the RAxML search. See Additional file 2 for details.

## Results and Discussion

### Many Avian Microinversions were Identified

Manual and automated searches revealed that non-coding regions associated with 11 of the 17 loci we examined contained microinversions (e.g., Figure 2) ranging from 5 bp to 38 bp (Additional file 2, Table S2). Their median length was 22 bp. A number of the microinversions identified here were much shorter than those reported in genome-scale comparisons of mammals [1,16], where the smallest microinversions were 23 bp and 31 bp, respectively. Although it is possible that birds and mammals have distinct microinversion size spectra, it seems more likely that the large-scale surveys of mammalian data failed to identify the shortest microinversions.

If λ_MI _was similar in birds and mammals, fewer than four microinversions would be expected given the amount of sequence data examined; instead, microinversions were identified at 49 positions (Table 1). Ma et al. [1] reported that short inversions are more common than long inversions. If this pattern continues as microinversions become even shorter than those they identified, the larger number of microinversions that we observed could reflect our identification of smaller inversions rather than any inherent difference between mammalian and avian genomes. The denser taxon sampling in our study, relative to whole genome studies in mammals, is also likely to have improved microinversion identification. Taken as a whole, our results suggest that previous studies that used mammalian data [1,6] underestimated λ_MI_.

The identification of microinversions can be difficult because point mutations and insertion-deletion events (indels) continue to accumulate after inversions. This has the potential to make ancient microinversions particularly difficult, or impossible, to identify. Denser taxon sampling can help by increasing the number of sequences closely related to those with the microinversion and by providing multiple versions of the inverted sequence (Additional file 2, Figure S1). Although the taxon sampling for this study was denser than previous surveys that used mammalian data, computational searches for microinversions were difficult. Many complementary strand alignments were not validated as actual inversions; the false positives reflected palindromes and other phenomena. bl2seq performed better than YASS, producing fewer false positives while still identifying all of the microinversions also found by YASS. However, even after employing two computational approaches, some microinversions were only identified "by eye" (Additional file 2, Table S2), suggesting that further improvements to the methods used to identify microinversions are required.

**Figure 3:**
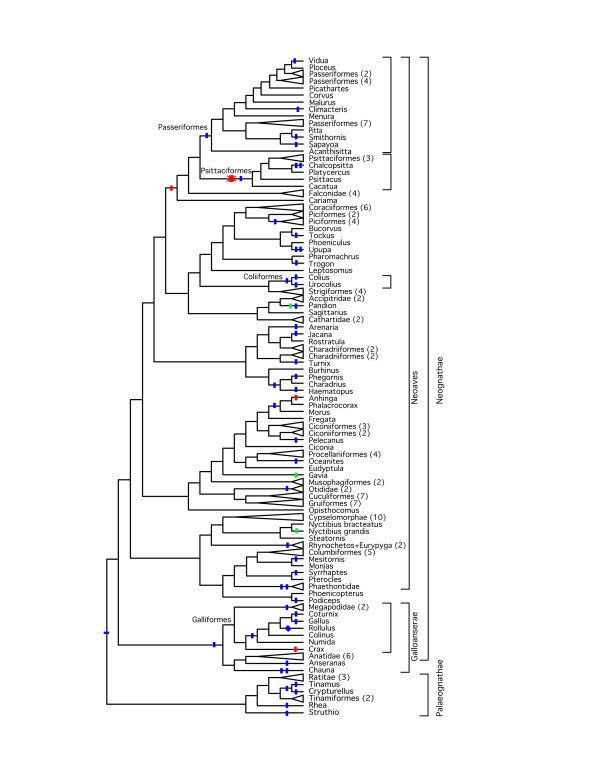
**Microinversions indicated on the Hackett et al**. [27]**phylogeny**. Inversions in introns are indicated with tick marks (blue for no homoplasy, green for the homoplastic inversions in *CLTC *intron 6, and red for the homoplastic inversions in *CLTC *intron 7). The 3' UTR inversion the *PCBD1*, which was obtained from selected galliform (see Results and Discussion), is indicated with a blue diamond. This mapping of character state changes assumes a reversal to the ancestral state in Psittaciformes for the *CLTC *intron 7 microinversion (indicated by an X over the red tick mark). An inversion in *CLTCL1 *where Palaeognathae and Neognathae differ is shown along the root branch. Orders united by microinversions are indicated using names above the branch uniting them and brackets to the right. The order Galliformes is emphasized because 3' UTRs were sequenced from additional taxa in that order (see text). This phylogeny is presented as a cladogram because many internal branches are very short and this presentation makes it easier to locate the inversion events. For branch length information refer to Figure 3 in Hackett et al. [27] and the chronogram presented for this publication (Additional file 2, Figure S3).

Most microinversions were assigned to terminal branches in the Hackett et al. [27] phylogeny (Figure 3) when the MP criterion was used. This raises the question of whether an acquisition bias caused us to miss a number of ancient microinversions that occurred closer to the base of the tree. However, the structure of the avian tree of life is dominated by a rapid radiation at the base of Neoaves, the most speciose avian supergroup (identified in Figure 3), leading to a tree dominated by terminal branches. Indeed, 70.8% of the overall treelength in the Hackett et al. ML tree [27] comprises terminal branches. The number of microinversions observed on terminal branches was not significantly different from expectation given the proportion of the tree that reflected internal and terminal branches (χ^2 ^= 3.0; *P *= 0.08). Thus, acquisition bias did not have a major impact upon our ability to identify ancient inversions.

### Avian Microinversion Rates Vary Among Loci

Estimates of λ_MI _differ among loci (Table 1). The Poisson model of microinversion accumulation (the null hypothesis) was rejected in favour of the NB model (which includes rate variation) using the LRT (2δ*ln*L = 27.55; *P *< 10^-6^). Excluding the highest-rate loci (*CLTC *and *CLTCL1*) eliminated our ability to reject the Poisson model (2δ*ln*L = 2.29; *P *= 0.13) and reduced the λ_MI _estimate to 0.25 microinversions Mb^-1 ^MY^-1 ^(the value presented in Figure 1; 95% confidence interval of 0.17 - 0.36). This suggests a "hotspot" model in which *CLTC *and *CLTCL1 *are inversion-prone. However, even the lower estimate of λ_MI _for "non-hotspot" loci greatly exceeded previous estimates of λ_MI_, consistent with our hypothesis that the identification of microinversions, especially the shortest inversions, has been improved relative to prior studies.

Surprisingly, both hotspot loci encode clathrin heavy chains, which are proteins critical for endocytosis [38], suggesting that the high microinversion rates could reflect their functional similarities. However, these clathrin heavy chain paralogs arose by duplication early in vertebrate evolution [39], and the homologous introns in *CLTC *and *CLTCL1 *do not exhibit detectable sequence similarity. Although specific intronic motifs can be overrepresented in functionally related genes [40], motifs common to the *CLTC *and *CLTCL1 *introns were not identified (data not shown). This suggests that it will be necessary to identify additional hotspot loci to understand the basis for inversion hotspots.

Microinversions were absent in some loci (Table 1), but it is unclear whether this reflects stochastic variation or the existence of "coldspots". 3' UTRs are coldspot candidates because they exhibit a lower rate of sequence evolution than introns [29,41] and they are known to include regulatory elements [42]. Many of these regulatory sequences are non-palindromic [43,44] and are unlikely to remain functional after inversion. Two to three microinversions were expected in our 3' UTR data (assuming equal rates for non-hotspot loci), but none were identified. We examined 3' UTRs from five additional loci (*ALDOB*, *CRYAA*, *EEF2*, *HMGN2*, and *PCBD1*), four of which have intronic microinversions (Table 1), by examining 23 members of the avian order Galliformes [41]. A 36 bp microinversion is present in the *Rollulus roulroul PCBD1 *3' UTR, indicating that these regions are not absolutely refractory to microinversions. Thus, future surveys should include 3' UTRs to improve λ_MI _estimates for those regions and establish whether they exhibit among-locus rate variation similar to introns.

### Homoplastic and Overlapping Microinversions Exist

Two microinversions in *CLTC *appeared homoplastic because the inverted forms were present in divergent lineages (e.g., Additional File 2, Figure S2). These homoplastic microinversions required at least three (*CLTC *intron 6) or four (*CLTC *intron 7) changes on the Hackett et al. [27] phylogeny using the MP criterion to explain the observed distribution of character states (Figure 3). Errors in the phylogeny are unlikely to explain this observation, since the relevant branches are well supported (compare Figure 3 to Figure 2 of Hackett et al. [27]; also see Additional File 2, Figure S2). Moreover, when these microinversions were mapped on other recent estimates of avian phylogeny using the MP criterion they require similar levels of homoplasy. These other estimates of phylogeny are based upon nuclear [26,45], mitochondrial [46-48], and morphological data [49,50], as well as expert opinion (e.g., Figure 27.10 in Cracraft et al. [51] and Figure 5 in Mayr [52]).

Hemiplasy is unlikely to explain the observed homoplastic microinversions for two reasons. First, hemiplasy would require maintenance of polymorphic inversions over multiple, long internal branches (estimates of branch lengths are presented as a chronogram in Additional File 2, Figure S3). Second, the estimate of the *CLTC *gene tree was not consistent with the microinversion distribution (Additional file 2, Figure S4), even in the single case in which branch lengths are short enough that hemiplasy is plausible. Thus, the *CLTC *inversions reflect genuine homoplasy, not hemiplasy, a novel finding for microinversions in animal nuclear genomes.

In addition to the homoplastic microinversions in *CLTC*, we also found several overlapping microinversions (Additional file 2, Table S2). All of these overlapping microinversions reflected independent inversions in distinct lineages. We identified two overlapping microinversions in *CLTC *and one in *CLTCL1*; the two overlapping microinversions in *CLTC *(INV-14 and INV-15; see Additional file 2, Table S2) also overlapped with one of the homoplastic microinversions in *CLTC *(INV-13). Thus, there were at least 12 inversion events in four specific regions of the two hotspot loci. There were also two additional overlapping inversions in low-rate loci (*EEF2 *and *IRF2*). Neither the homoplastic nor the overlapping microinversions were associated with stem-loop motifs (e.g., Additional file 2, Figure S4) or any other motifs that could be identified using MEME. These homoplastic and overlapping microinversions indicate that the actual state space for microinversions is likely to be smaller than their potential state space.

### Are Microinversions useful for Phylogenetics?

Although the existence of homoplastic microinversions demonstrates that they are not perfect characters, they still have the potential to be useful phylogenetic markers. The retention index of microinversions (RI_MI _= 0.949) given the Hackett et al. [27] tree is substantially higher than the retention index for nucleotide changes (RI_intron _= 0.52, RI_coding exon _= 0.54, RI_UTR _= 0.58). Such low amount of homoplasy suggests that an appropriate analytical approach (that accommodates homoplasy and hemiplasy) should yield an accurate species tree given a sufficient number of inversions.

Branches at the base of Neoaves are very short and this radiation is a classic example of a "bush" phylogeny [27]. In fact, the base of Neoaves has even been suggested to be a "hard" polytomy [53]. Hard polytomies reflect genuine multiple speciation events, so they cannot be represented as bifurcating trees. Even if Neoaves is a "soft" polytomy, many branches are likely to be <1 MY in length (Additional File 2, Figure S3; also see [26,45]). The low estimates of λ_MI _imply that microinversions will seldom occur along these short branches. How much sequence data would be necessary to resolve internodes of this length using microinversions? Power analysis assuming 1 MY branch lengths using the rate estimate that excludes the hotspot loci [54] indicates ~1.2 Mbp of non-coding sequence per taxon is needed to find at least one informative inversion and ~12 Mbp per taxon to identify an inversion on a specific branch (Additional file 2, Table S3). This estimate is orders of magnitude larger than the amount needed for of conventional analyses of sequence data (cf. Chojnowski et al. [26]). Moreover, it is desirable to identify multiple informative inversions along internodes given the potential for hemiplasy and homoplasy, suggesting that the use of microinversions as the sole source of information to estimate a phylogeny similar to the avian tree of life would require even more data (Additional file 2, Table S3).

### Microinversions and Multiple Sequence Alignment

The identification of microinversions is also important to ensure correct sequence alignment. Otherwise estimates of the amount of evolutionary change will be distorted, potentially resulting in incorrect phylogenetic estimation [19]. Algorithms for sequence alignment that include the possibility of inversions have been proposed [55-57], and they have the potential advantage of incorporating explicit penalties for inversion events. However, the optimal inversion penalty to limit false positives may be difficult to determine and the available algorithms are limited to the identification of non-overlapping microinversions. Overlapping microinversions were found at four loci that we examined, suggesting that the inability to identify overlapping inversions may represent a major limitation. Overlapping and homoplastic microinversions can be divided into three basic categories (Additional file 2, Figure S6), and the strategy we employed should be able to detect two of these categories efficiently. The third category (type III in Additional file 2, Figure S6, which corresponds to the case of multiple homoplastic or overlapping inversion events on a single branch) is expected to be rare. It may be possible to overcome this problem in a multiple sequence alignment framework using a divide-and-conquer approach by selecting subsets of taxa for which overlapping microinversions are less likely to be present. This would necessitate a subsequent assembly of the alignments. Moreover, such an approach might eliminate the benefits of dense taxon sampling. Despite these limitations, fully automated approaches could be less labour intensive than our approach. However, it is unclear whether microinversion identification can be fully automated since our results suggest that short microinversions may always require manual validation. Taken as a whole, these issues further emphasize the need to continue to improve algorithms for the detection and alignment of these interesting genomic changes.

## Conclusions

These analyses demonstrate that the identification of microinversions is important, despite the relatively low rate of accumulation of these genomic changes. This study revealed that microinversions accumulate more rapidly in avian genomes than expected based upon prior analyses of mammalian genomes, although this difference is likely to reflect the failure to identify very short inversions in the large-scale comparisons of mammalian data. If this failure to identify short microinversion does explain the differences among this and previous studies, the estimates of λ_MI _presented here, which are similar to the rate of accumulation of the most common type of avian TE insertion (Figure 1), may be more typical of vertebrate genomes. This likelihood that typical vertebrate λ_MI _values may be higher than suggested by previous studies emphasizes the importance of understanding the impact of microinversions upon genome evolution. We also documented the existence of microinversion hotspots, suggesting that some regions of the genome are especially prone to these mutations. The identification of additional hotspots may provide information about the mechanistic basis of these mutations. Indeed, we were able to exclude one proposed mechanism, the existence of conserved stem-loops, based upon an examination of the inversion hotspots identified here. Despite our observation that microinversions can exhibit homoplasy, they are still relatively reliable RGCs and as such may define gene tree bipartitions more accurately than conventional sequence data (see Nishihara et al. [58]). In the future, analytical methods that integrate microinversions with sequence data and information about other RGCs (and incorporate the potential for both hemiplasy and homoplasy) will facilitate robust resolution of difficult nodes in the tree of life and provide additional insights into the mechanism(s) responsible for their accumulation over evolutionary time.

## Authors' contributions

ELB designed the study, wrote many of the computer programs, conducted analyses, and wrote the manuscript. RTK helped design the study, validated computational microinversion searches, and helped draft the manuscript. K-LH manually identified the homoplastic microinversions and some overlapping microinversions. NRI-V contributed to analyses and wrote a novel computer program. AJB, JLC, JVS, RCKB, MJB, JH, SR, CCW, and TY helped to draft and revise the manuscript. All authors (except NRI-V) contributed to the sequence data analysed, helped construct and edit alignments, and manually searched the alignments for microinversions. All authors read and approved the final manuscript.

## Supplementary Material

Additional file 1 **Taxon list**. List of the taxa used for this analysis and the accession numbers for the novel *CLTCL1 *sequences collected for this study, in Microsoft Excel format. Click here for file

Additional file 2 **Supplementary information**. Six figures, three tables, and supplementary methods (including the details of the literature survey used to estimate the rates of various types of genomic changes and the power analysis described in the main text), in pdf format. Click here for file

Additional file 3 **Details of a microinversion search**. An example of a microinversion search (of *TPM1 *intron 6) is presented along with a description of the search algorithm using pseudocode, in Microsoft Excel format. Click here for file
