# Supplementary material for: Homoplastic microinversions and the avian tree of life
Source: BMC Evol Biol. 2011 May 25;11:141. doi: 10.1186/1471-2148-11-141 (PMC3123225; doi:10.1186/1471-2148-11-141)
Supplement: Additional file 2 — Supplementary information. Six figures, three tables, and supplementary methods (including the details of the literature survey used to estimate the rates of various types of genomic changes and the power analysis described in the main text), in pdf format. [file 1471-2148-11-141-S2.PDF]

Supplementary Information for Braun et al. "Homoplastic Microinversions and the Avian Tree of Life"

Supplementary methods

Evolutionary rate estimates for Figure 1

Although a comprehensive meta-analysis to examine the rates at which various genomic changes accumulate is beyond the scope of the present work, it is important to place estimates of the rate of accumulation for relatively poorly characterized genomic changes in the context of rates that are more familiar. For that reason, we reviewed the relevant literature to obtain approximate estimates of these rates. The Chaisson et al. (2006) estimate of the mammalian microinversion rate (λ_{MI}) was ~0.015 microinversions per megabase per million years (microinversions Mb^{-1} MY^{-1}). Although Chaisson et al. (2006) did not present detailed information regarding the size spectrum of the microinversions they identified, making comparisons to other studies difficult, these units (genomic changes Mb^{-1} MY^{-1}) seem reasonable to use for comparisons. These units were also used for genomes that are <1 Mb in size (e.g., chloroplast genomes).

Nucleotide substitutions are the most rapidly accumulating genomic changes that were used for this comparison. The neutral nucleotide substitution rate is often assumed 0.005 substitutions $site^{-1}$ MY^{-1} in mammals (e.g., Penny et al. 2001). This value is slightly higher than estimates of the neutral nucleotide substitution rate that Chojnowski et al. (2008) obtained for birds using introns (0.0014 substitutions $site^{-1}$ MY^{-1}). Gaut (1998) reviewed the available estimates of angiosperm nucleotide substitution rates, which ranged from 0.005-0.03 substitutions $site^{-1}$ MY^{-1} for nuclear substitutions (a "consensus" value of 0.015 substitutions $site^{-1}$ MY^{-1} was chosen for simplicity) and 0.001-0.003 substitutions $site^{-1}$ MY^{-1} for chloroplast substitutions (a consensus value of 0.002 substitutions $site^{-1}$ MY^{-1} was chosen). To convert these rates to the appropriate units for comparison (nucleotide substitutions Mb^{-1} MY^{-1}) rate estimates were multiplied by 10^6.

Estimates of the mammalian non-coding region indel rate are ~1/8 of the nucleotide substitution rate (Lunter 2007). This suggests a rate of ~625 indels Mb^{-1} MY^{-1}. The avian non-coding indel rate was extrapolated from Bonilla et al. (2010) who used ~4.8 kb of non-coding DNA for a 23 taxon tree. MP analysis of a dataset generated using the simple coding method of Simmons & Ochoterena (2000) revealed a total of 902 indel events. Combining this value with the sequence length and *TL* (604 MY; obtained as described below) allowed us to estimate a rate of ~300 indels Mb^{-1} MY^{-1} for avian non-coding regions. It is unclear whether this difference reflects the use of methods used to estimate the indel rate for non-coding regions, differences among the regions of the genome examined by these studies, or differences between birds and mammals. Regardless, these estimates suggest typical vertebrate non-coding indel rates are approximately an order of magnitude less than the neutral nucleotide substitution rate.

The mammalian coding region indel rate was obtained using information from Murphy et al. (2007), who reported 5,648 and 5,723 coding region indels in human-armadillo and human-elephant comparisons, respectively. Combining the amount of sequence data examined (17-19 Mb) and the divergence time estimate (105 MYA) from Murphy et al. (2007) suggests a rate of ~1.5 indels Mb^{-1} MY^{-1} coding region. We could not obtain a similar estimate using avian data.

Rates of accumulation for transposable element (TE) insertions show substantial variation that reflects the specifics of elements that are being considered. For simplicity, we limited

consideration to the accumulation of the most common avian TE (the CR1 element) because it was possible to use the same methods and data matrix that we use to estimate λ_{MI}. Han et al. (2011) identified 59 CR1 insertions in an alignment similar to the alignment used for this study. Since the sequence length and time available for TE insertions in the Han et al. (2011) study are similar to those for this study the CR1 insertion rate is ~0.4 TE insertions Mb^{-1} MY^{-1}.

Because Chaisson et al. (2006) did not present information regarding their microinversion size we used the Ma et al. (2006) to obtain an a second *a priori* estimate of λ_{MI} for vertebrate nuclear genomes. Ma et al. (2006) identified 12,057 microinversions in complete genome comparisons involving four mammals and they presented a size spectrum that overlaps with the sizes microinversions we obtained. Combining this number of microinversions with divergence times estimates from Murphy et al. (2007) suggests an estimate of λ_{MI} (~0.018 microinversions Mb^{-1} MY^{-1}) very similar the estimate from Chaisson et al. (2006). Most (6,277) of the microinversions identified by Ma et al. (2006) were identified when the two rodents in the study (rat and mouse) were compared, suggesting λ_{MI} could be higher in murid rodents (~0.065 microinversions Mb^{-1} MY^{-1}). Several other types of substitutions accumulate more rapidly in rodents than in other mammalian lineages (e.g., Cooper et al. 2003), making it is reasonable to speculate that λ_{MI} is also higher in this lineage. However, it remains possible that λ_{MI} has been underestimated in the other mammalian lineages that Ma et al. (2006) examined and it is important to recognize that there is no direct evidence that λ_{MI} is correlated with the rates of accumulation for other types of genomic changes in mammals or any other vertebrate lineage.

Although the microinversion size spectra presented in some prior studies (Feuk et al. 2005; Ma et al. 2006) overlaps with the sizes of microinversions we identified, it seems likely that these large-scale studies did not identify the smallest inversions. Taken as a whole, our analyses of avian microinversions suggest that the relatively low λ_{MI} estimates based upon Chaisson et al. (2006) and Ma et al. (2006) reflect this acquisition bias. Thus, it may be more appropriate to view the estimates of λ_{MI} from those studies as the rate of accumulation for longer microinversions (>30 bp based upon the size spectra reported by Ma et al. 2006).

We also extended our estimates of λ_{MI} to angiosperm chloroplast DNA by using information about a 1.7 kb region sequenced from ten *Jasminum* species (and one *Monodora* outgroup) where five inversion events were found (Kim & Lee 2005). *TL* for that was ~135 MY based upon examination of branch lengths and a molecular clock analyses of the same group (Lee et al. 2007). This suggests the angiosperm chloroplast λ_{MI} is ~22 microinversions Mb^{-1} MY^{-1}, although this rate should be viewed as approximate given the relatively small number of microinversions that were identified.

Several different types of genomic changes have been shown to accumulate at different rates in different parts of the genome. For example, regional variation has been documented for both neutral nucleotide substitution rates (e.g., Webster et al. 2004) and non-coding indel rates (Lunter 2007). Likewise, there are many examples of among-taxon variation in the evolutionary rate within groups; Figure 3 in Hackett et al. (2008) provides an excellent example of this rate variation using a subset of the data that were analyzed for this study. Our analyses of λ_{MI} in birds also indicate that there is among-locus variation in the rate of accumulation for these changes (see main text). λ_{MI} may also exhibit among-taxon variation, although the limited number of inversions observed in this study make difficult to establish the magnitude of this variation or even whether this variation actually exists. However, variation among-taxa and among-loci is

typically less than an order of magnitude suggesting that the rate estimates presented here provide a sufficient framework to understand the rates at which different genomic changes accumulate over evolutionary time (summarized in Figure 1 in the main text).

Avian microinversion rate estimates

To calculate λ_{MI} in birds, we estimated the number of inversion events using the MP criterion and combined that information with the mean length of the non-coding data examined (in Mb) and the amount of time available for microinversions to accumulate. Time available for inversions was estimated using the treelength (*TL*) of the Hackett et al. (2008) tree in MY, estimated using an ultrametric version of that tree generated by non-parametric rate smoothing (NPRS; Sanderson 1997). Since there was missing data for specific taxa in some partitions, *TL* for each locus may differ. Locus-specific *TL* values were calculated using the ultrametric Hackett et al. (2008) tree after pruning branches that correspond to missing taxa.

Divergence times on the ultrametric tree were calculated by assuming the basal split in Neoaves occurred 100 MYA, a value consistent with the higher estimates obtained in molecular clock studies (e.g., Brown et al. 2008). The timing of evolutionary divergences in birds remains controversial (Brown et al. 2007; Ericson et al. 2007; Chojnowski et al. 2008) and we view the value of *TL* based upon the 100 MYA Neoaves origin calibration as a reasonable upper limit for the time available for mutation. An approximate lower limit can be obtained by constraining the divergence of *Anseranas* and the Anatidae to be 68 MYA based upon the fossil *Vegavis iaai* (Clarke et al. 2005). For the purposes of this study, the more ancient calibration is conservative since it is not expected to overestimate λ_{MI} (potentially an important consideration given the relatively high estimates of that λ_{MI} we obtained). To convert microinversion rate estimates based upon the 100 MYA calibration to those based upon the *Vegavis* constraint (which implies that the origin of Neoaves was 85.4 MYA) they should be multiplied by a constant (1.17).

A similar approach was used to generate an ultrametric tree based upon the Bonilla et al. (2010) galliform phylogeny. Briefly, the tree presented as Figure 2 in Bonilla et al. (2010) was rooted between Megapodidae and the remaining Galliformes, as suggested by Hackett et al. (2008) and a number of additional studies (e.g., Crowe et al. 2006). This tree was then subjected to NPRS (Sanderson 1997) and *TL* was calculated by assuming the divergence between the families Cracidae and Phasianidae occurred 68.8 MYA. This calibration was based upon the ultrametric Hackett et al. (2008) tree described above. Both ultrametric trees are available from the Early Bird website (http://www.biology.ufl.edu/earlybird) or upon request from ELB.

Among-locus microinversion rate variation

We examined the rate of microinversion accumulation by comparing the simplest evolutionary model (a Poisson process) to the more complex negative binomial (NB) model, which allows rate variation across loci, as described by Han et al. (2011). The ML estimate of λ_{MI} given the Poisson model for a specific locus of length *Len* given *k* observed microinversions and a taxon sample corresponding to treelength (*TL*) is simply *k*/(*Len* x *TL*). The likelihood is proportional to the probability of observing *k* inversions given λ_{MI}, *Len*, and *TL*, which can be calculated using equation 1:

$$P\left(k \mid \lambda_{MI}, Len, TL\right) = \frac{\left(\lambda_{MI}\left[Len \times TL\right]\right)^{k} e^{-\lambda_{MI}\left[Len \times TL\right]}}{k!} \tag{1}$$

The likelihood given multiple loci is simply the product of the likelihoods for individual loci. The NB model is similar, but it adds a non-negative variance inflation parameter (c) to the other variables used in equation 1 as shown below:

$$P\left(k \mid \lambda_{MI}, Len, TL, c\right) = \frac{\left(\lambda_{MI}\left[Len \times TL\right]\right)^{k}}{k!} \times \frac{\Gamma\left(1/c + k\right)}{\Gamma\left(1/c\right)\left(\lambda_{MI}\left[Len \times TL\right] + 1/c\right)^{k}} \times \left(1 + \frac{\lambda_{MI}\left[Len \times TL\right]}{1/c}\right)^{-1/c} \quad (2)$$

The likelihood ratio test is straightforward since the NB and Poisson models differ by a single parameter (equation 2 reduces to equation 1 when $c = 0$). This allows us to compare the null hypothesis of equal microinversion rates of at different loci to the alternative hypothesis of variable rates of microinversions at different loci.

Power analyses

Determining how many data are necessary to yield a satisfactory estimate of a phylogeny has been examined using at least two distinct ways (reviewed by Spinks et al. 2009). The first set of approaches is based upon empirical data, ranging from simple brute force (i.e., continue data collection until one achieves a specified degree of precision or accuracy) to extrapolation from existing data matrices (e.g., the "pseudo-bootstrap" method; DeFilippis & Moore 2000). The small number of microinversions available for phylogenetic studies makes empirical approaches difficult. The other set of approaches to this question involves the use of evolutionary models to predict the amount of data necessary to achieve a specific degree of precision or accuracy, using either analytical approaches (e.g., Walsh et al. 1999; Braun & Kimball 2001) or simulations (e.g., Chojnowski et al. 2008; Spinks et al. 2009). This approach is also difficult for microinversions due to the limited amount of information regarding the appropriate model to use (see main text), but it is possible to place some limits on amount that one would need to collect using the simple power analysis of Braun & Kimball (2001) .

Braun & Kimball (2001) pointed out that the probability of obtaining at least one synapomorphy along a short internal branch is straightforward to calculate using equation 1 (above) if one assumes the relevant characters accumulate according to a Poisson model of evolution. A similar calculation is possible for the likelihood of finding some minimum number of genomic changes (e.g., three or more) on a branch of specified length. In this case, the complement of the probability of finding fewer substitutions is used (i.e., the sum of the probabilities given zero, one, or two substitutions). This approach was used to establish the amount of sequence data needed to be 95% certain that the specified number of microinversions could be identified.

The Braun & Kimball (2001) power analysis treats internal branch lengths in a phylogenetic tree as the effect size. This method assumes that homoplasy is rare and that the simplest evolutionary model (a Poisson process) provides an adequate description of the evolutionary process, and it focuses on establishing the sequence length necessary to have a specific probability (e.g., 95%) of finding at least one genomic change (i.e., microinversion) that occurred on a relevant internal branch. Despite the evidence that some microinversions exhibit homoplasy, the assumption that homoplastic microinversion is relatively rare seems justified. The use of a Poisson model may seem problematic given the better fit of the NB model, but our inability to reject a Poisson model once the hotspot loci (*CLTC* and *CLTCL1*) were removed suggests that using the simpler model is justified.

A reasonable set of target branches for this power analysis can be obtained by considering the relationships that were poorly supported in Hackett et al. (2008). These relationships were summarized as an incompletely resolved tree (Figure 4 in Hackett et al. 2008) that would require the addition of ten internal branches for complete resolution. Thus, a reasonable lower limit on the amount of non-coding sequence data necessary to provide useful information regarding these branches can be provided by calculating the probability of finding at least one inversion along any one of those (currently unresolved) branches. Estimates of the amounts of sequence data necessary given different assumptions regarding the lengths of the internal branches are provided in Table S3 in the column labeled "≥1 informative". If one is interested in the amount of data needed to have the same probability of obtaining at least one microinversion along a specific short branch the sequence lengths in Table S3 should be multiplied by ten.

Hemiplasy is also a problem for analyses of microinversions, especially for the shortest branches presented in Table S3. Thus, it is desirable to obtain multiple microinversions along specific branches before making firm conclusions regarding relationships. For this reason the amount of data necessary to have a 95% probability of obtaining at least three microinversions along a specific short branch is also presented. Estimating the exact amounts of data necessary under these conditions is relatively complex, since it depends upon both the model of microinversion accumulation and demographic factor that determine the probability of hemiplasy. Thus, we view the estimates in Table S3, which can be as large as 20% of avian genomes, as approximate guidelines.

Supplementary references

Supplementary references are listed alphabetically and references that were also used in the main text are indicated with an asterisk.

*Bonilla et al. 2010 *see main text.*

*Braun & Kimball 2001 *see main text.*

Brown JW, Payne RB, Mindell DP: **Nuclear DNA does not reconcile 'rocks' and 'clocks' in Neoaves: a comment on Ericson et al.** *Biol Lett* 2007, **3:**257-259.

*Brown et al. 2008 *see main text.*

*Chaisson et al. 2006 *see main text.*

*Chojnowski et al. 2008 *see main text.*

Clarke JA, Tambussi CP, Noriega JI, Erickson GM, Ketcham RA: **Definitive fossil evidence for the extant avian radiation in the Cretaceous.** *Nature* 2005, **433:**305-308.

*Cooper et al. 2003 *see main text.*

Crowe TM, Bowie RCK, Bloomer P, Mandiwana TG, Hedderson TAJ, Randi E, Pereira SL, Wakeling J: **Phylogenetics, biogeography and classification of, and character evolution in, gamebirds (Aves: Galliformes): effects of character exclusion, data partitioning and missing data.** *Cladistics* 2006, **22**:495-532.

DeFilippis VR, Moore WS: **Resolution of phylogenetic relationships among recently evolved species as a function of amount of DNA sequence: an empirical study based on woodpeckers (Aves: Picidae).** *Mol Phylogenet Evol* 2000, **16:**143-160.

Ericson PGP, Anderson CL, Mayr G: **Hangin' onto our rocks 'n clocks: A reply to Brown et al.** *Biol Lett* 2007 **3:**260-261.

Gaut BS: **Molecular clocks and nucleotide substitution rates in plants.** In *Evolutionary Biology. Volume* 30. Edited by Hecht MK, MacIntyre RJ, Clegg MT. New York: Plenum Press; 1998: 93-120.

*Hackett et al. 2008 *see main text*.

*Han et al. 2011 *see main text*.

*Kim & Lee 2005 *see main text*.

Lunter G: **Probabilistic whole-genome alignments reveal high indel rates in the human and mouse genomes.** *Bioinformatics* 2007 **23:**i289-i296.

Murphy WJ, Pringle TH, Crider TA, Springer MS, Miller W: 2007 **Using genomic data to unravel the root of the placental mammal phylogeny.** *Genome Res* 2007, **17:**413-421.

Penny D, McComish BJ, Charleston MD, Hendy MD: **Mathematical elegance with biochemical realism: The covarion model of molecular evolution**. *J Mol Evol* 2001, **53:**711-723.

Sanderson MJ: **A nonparametric approach to estimating divergence times in the absence of rate constancy.** *Mol Biol Evol* 1997, **14:**1218–1231.

Simmons MP, Ochoterena H: **Gaps as characters in sequence-based phylogenetic analysis.** *Syst Biol* 2000, **49:**369-381.

Spinks PQ, Thomson RC, Lovely GA, Shaffer HB: **Assessing what is needed to resolve a molecular phylogeny: simulations and empirical data from emydid turtles.** *BMC Evol Biol* 2009, **9:**56.

*Stamatakis 2006 *see main text*.

Walsh HE, Kidd MG, Moum T, Friesen VL: **Polytomies and the power of phylogenetic inference.** *Evolution* 1999, **53:**932-937.

Webster MT, Smith NGC, Lercher MJ, Ellegren H: **Gene expression, synteny, and local similarity in human noncoding mutation rates.** *Mol Biol Evol* 2004, **21:**1820-1830.

Zuker M: **Mfold web server for nucleic acid folding and hybridization prediction.** *Nucleic Acids Res* 2003, **31:**3406-3415.

(*a*)

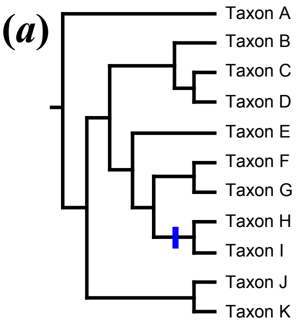

Microinversion uniting taxa H and I

(*b*)

	Group 1							Group 2		Group 1	
	**A**	**B**	**C**	**D**	**E**	**F**	**G**	**H**	**I**	**J**	**K**
**A**											
**B**	-										
**C**	-	-									
**D**	-	-	-								
**E**	-	-	-	-							
**F**	-	-	-	-	-						
**G**	-	-	-	-	-	-					
**H**	+	++	++	++	+++	++++	++++				
**I**	+	++	++	++	+++	++++	++++	-			
**J**	-	-	-	-	-	-	-	+	+		
**K**	-	-	-	-	-	-	-	+	+	-	

Matrix of pairwise complementary strand alignments

Figure S1 - Microinversion search strategy using complementary strand alignments.

Hypothetical example of the microinversion search strategy we used. (*a*) Cladogram showing a microinversion uniting taxa H and I. Informative microinversions will define two groups of taxa, the outgroup taxa (group 1) and members of the clade united by the microinversion (group 2). (*b*) Matrix showing the taxa with the potential to exhibit detectable pairwise complementary strand alignments. Divergence among taxa due to subsequence point mutations and indels is expected to erode sequence similarity in the inverted region; the expected "strength" of the complementary strand alignments is indicated using qualitative values ('+' to '++++'). These values are included to provide an approximate guideline. Specific mutations (e.g., deletions) have the potential to render some microinversions undetectable in closely related taxa even when they remain detectable when more distantly related taxa are compared.

Homoplastic microinversions in *CLTC* intron 6

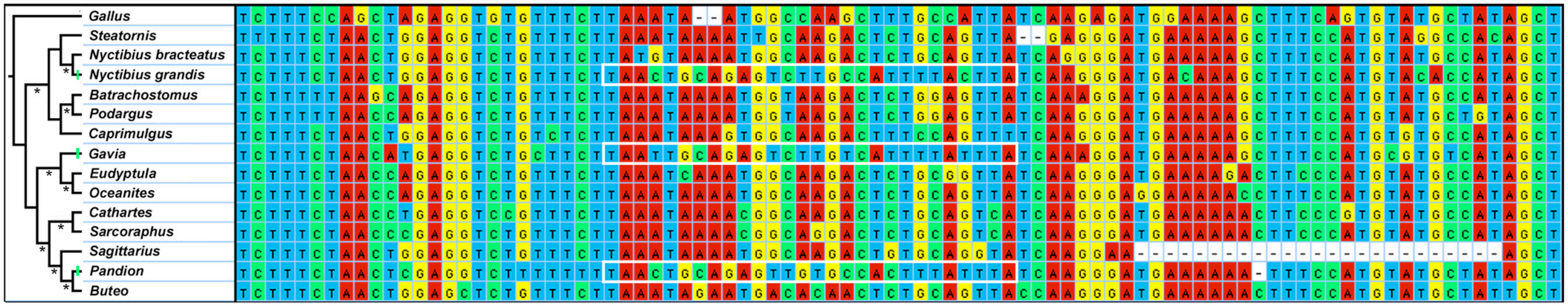

Figure S2 - Multiple sequence alignment showing the homoplastic microinversions in *CLTC* intron 6.

Inverted sequences are outlined in white and phylogenetic relationships based upon Hackett et al. (2008) are shown to the left of the alignment with well-supported branches (defined as those with ML bootstrap support ≥80% in the Hackett et al. [2008] study) indicated with stars below the relevant branch. Following Figure 3 in the main text, inversion events are indicated on the phylogeny using green lines. Support for the branches separating the taxa with microinversions in the *CLTC* gene tree are presented below in Figure S2*b*.

(*a*)

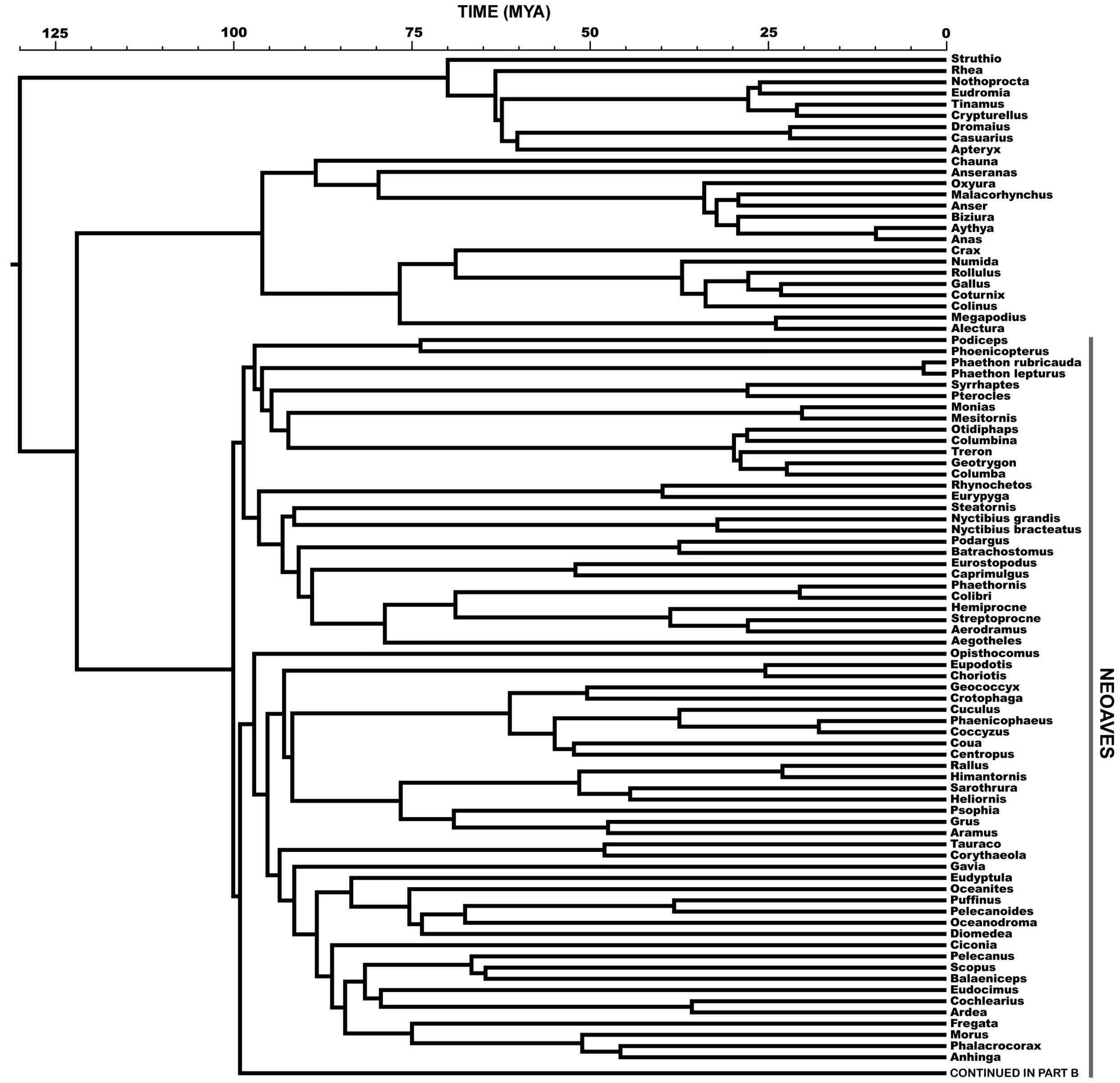

Figure S3 - Chronogram based upon the Hackett et al. (2008) phylogeny.

An ultrametric version of avian phylogeny presented as Figure 3 in Hackett et al. (2008) with branch lengths that reflect non-parametric rate smoothing (Sanderson 1997) of the ML branch length estimates. The time scale was calibrated by assuming the basal split in Neoaves occurred 100 MYA. This figure is divided into two parts: (*a*) Paleognathae, Galloanserae, and part of Neoaves; and (*b*) the remainder of Neoaves. This chronogram is available on the Early Bird website (http://www.biology.ufl.edu/ earlybird) in nexus format or upon request from ELB.

(*b*)

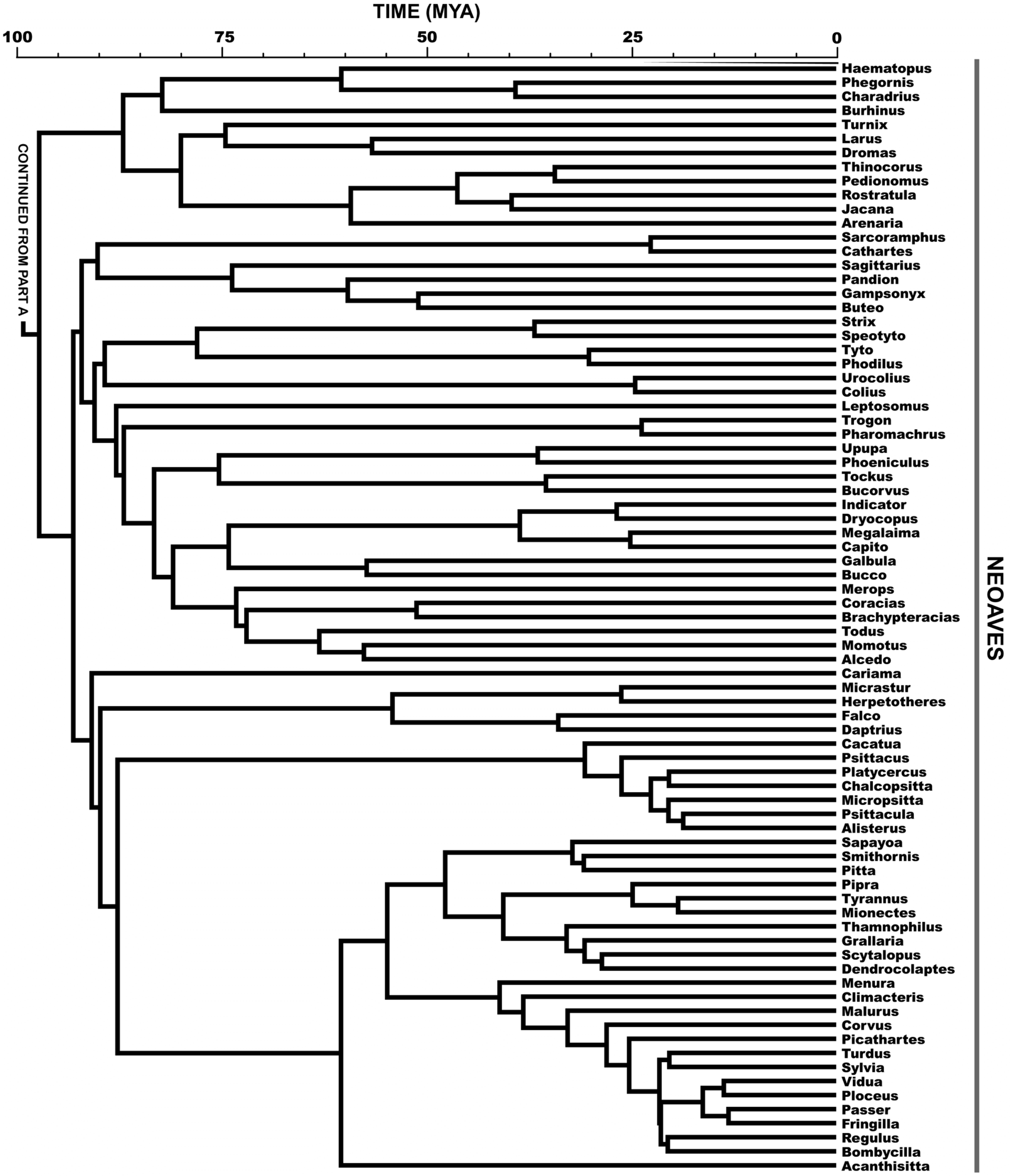

Figure S3 - Chronogram based upon the Hackett et al. (2008) phylogeny, continued.
Approximate divergence times for a subset of Neoaves (shorebirds [Charadriiformes] and the Hackett et al. [2008] landbird clade) are shown. See Table S1 for taxonomic details; additional information about this chronogram is presented in the Figure S3*a* legend (previous page).

(a)

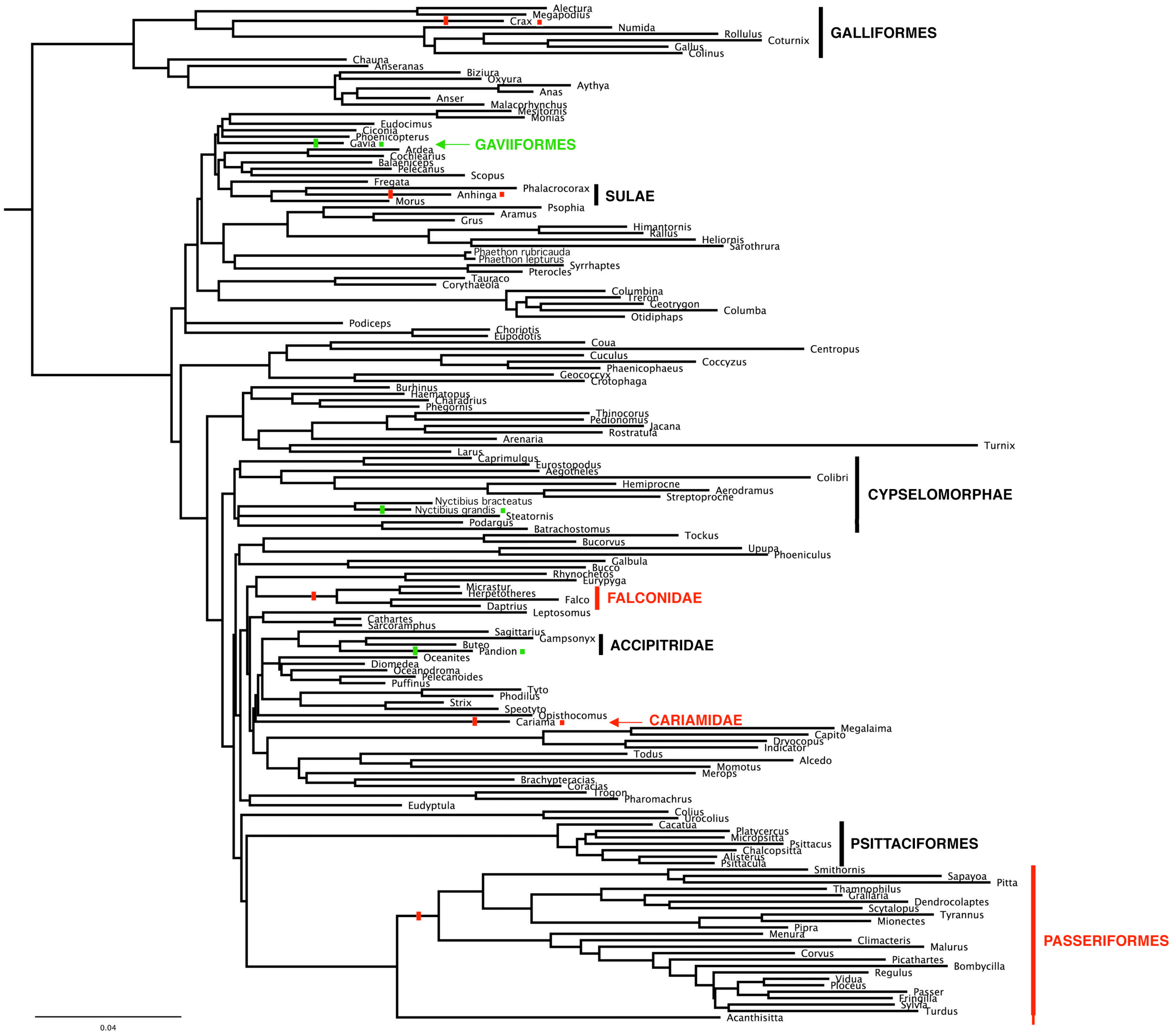

Figure S4 - The *CLTC* gene tree indicates the homoplastic microinversions are unlikely to reflect hemiplasy.

Estimate of *CLTC* gene tree (-*ln* L = 75153.652482) obtained using the GTR+Γ model in RAxML (Stamatakis 2006) with 25 randomized starting tree searches. (*a*) Phylogram showing relative branch lengths with the homoplastic microinversion indicated in green (*CLTC* intron 6) and red (*CLTC* intron 7). The names of larger clades are presented in color if all members have the inverted form of the sequence; otherwise they are presented in black. The character state for the deepest-branching member of Passeriformes (*Acanthisitta chloris*) is unclear because the relevant region of *CLTC* intron 7 is covered by large deletion. For simplicity, the inversion event is presented on the branch uniting all other Passeriformes. Note that this gene tree requires five inversion events in *CLTC* intron 7 instead of the four events required on the Hackett et al. (2008) topology (see Figure 3). (*b*) Cladogram showing ML bootstrap support for groups in the *CLTC* gene tree (next page).

(b)

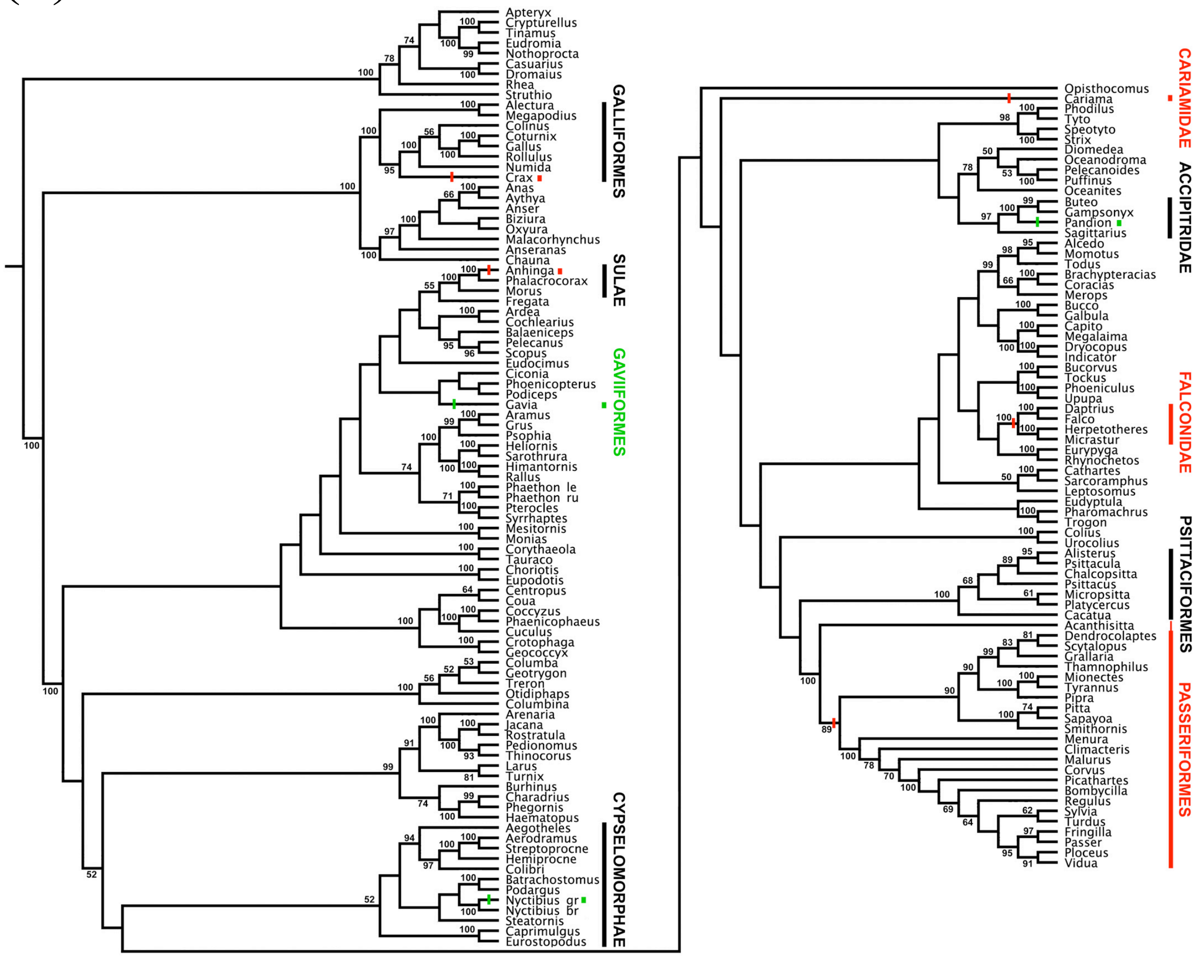

Figure S4 - The *CLTC* gene tree indicates the homoplastic microinversions are unlikely to reflect hemiplasy, continued.

(*b*) ML bootstrap consensus tree for *CLTC* presented as a cladogram. Bootstrap support (as a percentage of 500 replicates) is presented alongside branches, with values <50% omitted. Microinversions are labeled on this tree in the same way they are in Figure S4*a* (previous page).

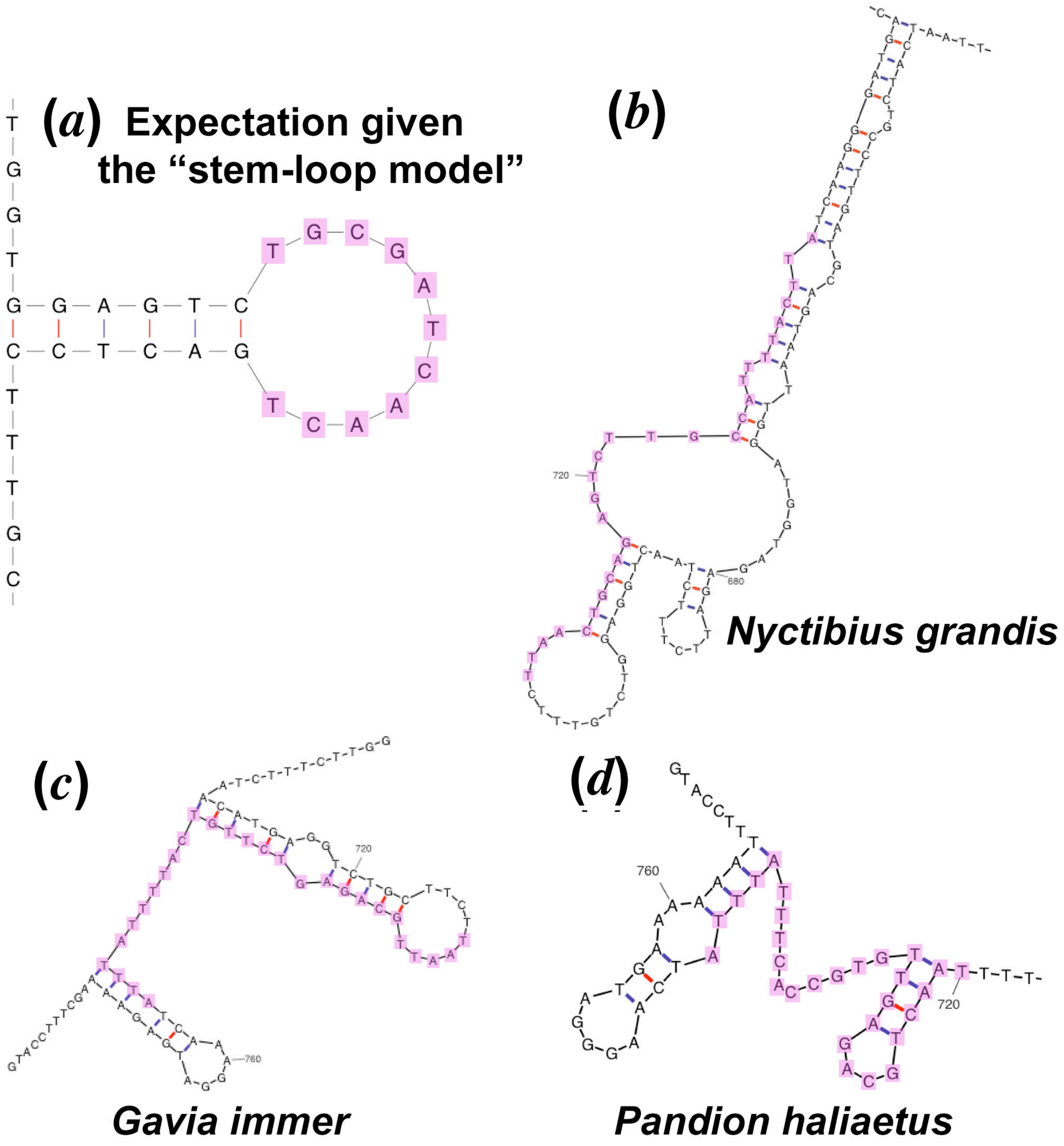

Figure S5 - The homoplastic microinversions in *CLTC* do not appear to be associated with conserved stem-loop structures.

(*a*) Expected position of an inverted sequence (highlighted) given the stem-loop model (e.g., Kim and Lee 2005), which predicts that homoplastic microinversions are associated with conserved stem-loop. DNA mfold (http://mfold.bioinfo.rpi.edu/cgi-bin/dna-form1.cgi; Zuker [2003]) did not reveal stem-loop structures in *CLTC* intron 6 of the (*b*) Great potoo (*Nyctibius grandis*), (*c*) Common loon (*Gavia immer*), or (*d*) Osprey (*Pandion haliaetus*). The structures presented here emphasize both the absence of conserved folding structures and the lack of correspondence between any possible stem-loops and the inversions in *N. grandis*, *Gavia*, and *Pandion*. Alternative folding structures predicted by mfold showed a similar lack of correspondence with the position of the inversion. Similar results were obtained using the other homoplastic microinversion and the overlapping microinversions (data not shown).

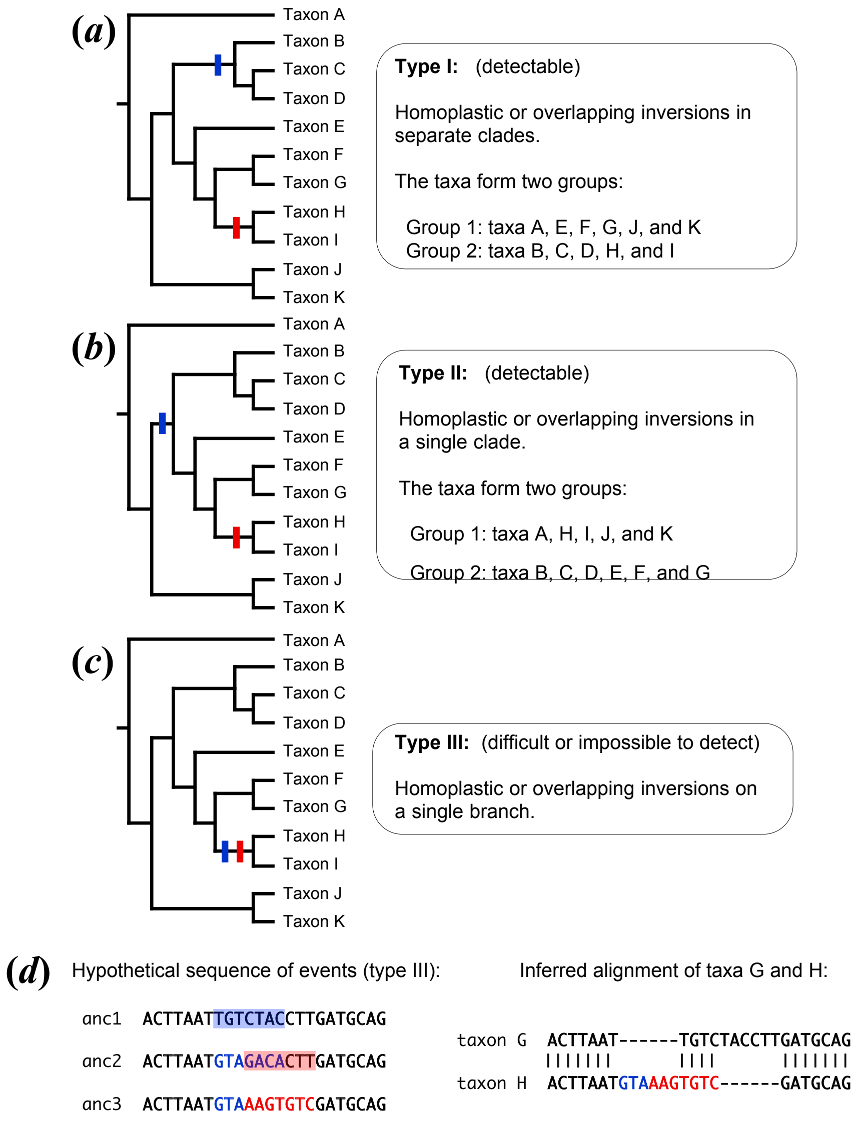

Figure S6 - Homoplastic and overlapping microinversions.

Homoplastic and overlapping microinversions can be divided into three categories, two of which can be detected using the strategy outlined in the Methods. Both type I (*a*) and type II (*b*) mciroinversions divide the taxa into at least two groups (similar to the groups in Figure S1). There may be three groups if the overlapping region is short. With the exception of INV-13 (Table S2), where the most parsimonious reconstruction for Passeriformes, Psittaciformes, Falcondiae, and Cariamidae conforms to a type II distribution, all homoplastic and overlapping microinversions that we identified had a type I distribution. (*c*) Type III microinversions are undetectable unless the overlap if very short, although they are expected to be rare given the low estimates of λ_{MI}. (*d*) Example showing the impact that overlapping microinversions might have upon sequence alignment. Inversions are indicated in this example by shading the affected nucleotides (colors indicate the nucleotides the inverted during each events indicated in part *c*). This could render the microinversion undetectable even in the absence of point mutations and indels; additional mutations will further complicate the detection of these microinversions. Type II microinversions may appear to be type III microinversions when taxon sampling is sparse.

Table S1 - Taxa used for this analysis and accession numbers for novel sequences.

This table is presented as an excel spreadsheet (Additional File 1).

Table S2 - Microinversions identified by this study.

A complete list of the microinversions identified is presented below in landscape format.

Table S3 - Power of microinversions to define short internal branches.

Branch Length[c] (MY)	Amount of Sequence Data Needed (in Mb)[a] $\lambda_{MI} = 0.25$[b] ≥1 informative[d]	≥3 on a specific branch[e]	$\lambda_{MI} = 0.015$[b] ≥1 informative[d]	≥3 on a specific branch[e]
0.1	12	252	200	—[f]
0.2	6	126	100	—[f]
0.3	4	84	67	—[f]
0.4	3	63	50	—[f]
0.5	2.4	50	40	839
0.6	2	42	33	700
0.7	1.7	36	29	600
0.8	1.5	31	25	525
0.9	1.3	28	22	466
1	1.2	25	20	420
1.25	0.96	20	16	336
1.5	0.8	17	13	280
1.75	0.68	14	11	240
2	0.6	13	10	210

[a] This table presents the amount of sequence data needed to be 95% certain that the listed number of microinversions will be found on internal branch.

[b] The microinversion rate (λ_{MI}) presented as inversions Mb^{-1} MY^{-1}. Estimates of λ_{MI} used are 0.25 (from this study) and 0.015 (from Chaisson et al. 2006). The lower Chaisson et al. (2006) rate appears to reflect the failure to identify very short inversions, so it should be viewed as an estimate for the rate of longer microinversions (those longer than ~30 bp based upon the Ma et al. [2006] size spectrum).

[c] Length (in millions of years) assumed for the short branches in the avian tree of life.

[d] Amount of sequence data needed to have a 95% probability of finding at least one parsimony informative microinversion on a short internal branch in the avian tree of life. The amount of data needed to identify at least one microinversion on a specific short branch is 10-fold greater.

[e] Amount of sequence data needed to have a 95% probability of finding at least three parsimony informative microinversions on a specific short internal branch in the avian tree of life

[f] The amount of sequence data necessary to meet this criterion exceeds the size of some avian genomes.

Table S2 - Microinversions identified by this study.

INV #	Locus	Intron	Length (bp)[a]	Inverted sequence found in:	Identified bl2seq	Identified "by eye"	Notes
ALDOB – (total of 4 sites with inversions)							
1	*ALDOB*	3	10	*Vidua*	no	yes	Sequence is relatively divergent.
2	*ALDOB*	5	6	Psittaciformes	no	yes	
3	*ALDOB*	6	11	*Podiceps*	no	yes	
4	*ALDOB*	7	6	*Smithornis*	no	yes	
CLTC – (total of 14 sites[b] with inversions)							
5	*CLTC*	6	19-22	*Capito*, *Megalaima*, *Indicator*, *Dryocopus*	yes	no	
6	*CLTC*	6	19	Phaethontidae	yes	no	
7	*CLTC*	6	38	*Anseranas*	yes	yes	
8	*CLTC*	6	24	*Chauna*	yes	no	Overlaps with INV-9.
9	*CLTC*	6	29	Phaethontidae	yes	no	Overlaps with INV-8.
10	*CLTC*	6	37	*Pelecanus*	yes	yes	
11	*CLTC*	6	28	*Nyctibius grandis*, *Gavia*, *Pandion*	yes	yes	Homoplastic[b] (see text).
12	*CLTC*	7	24	Coliiformes	yes	no	
13	*CLTC*	7	19-29	*Crax*, *Anhinga*, *Cariama*, Falconidae, Passeriformes	yes	yes	Homoplastic[b] (see text) and overlaps with INV-14 and INV-15. Character state unknown in *Acanthisitta* (Passeriformes) due to large deletion in this region.
14	*CLTC*	7	29-30	*Crypturellus*, *Tinamus*	yes	yes	Overlaps with INV-13 and INV-15.
15	*CLTC*	7	34	*Oceanites*	yes	yes	Overlaps with INV-13 and INV-14.
16	*CLTC*	7	27-39	Galloanserae (Galliformes and Anseriformes)	yes	no	
17	*CLTC*	7	30-31	Megapodidae (*Alectura*, *Megapodius*)	yes	no	
18	*CLTC*	7	20-28	Passeriformes	yes	no	

[a] The range of observed lengths for the inverted sequence is reported; this value is not an estimate of the ancestral length of the microinversion.
[b] Microinversions were considered to have occurred at the same site only if both endpoints of the inversion appeared identical based upon the length of the relevant complementary strand alignments.

Table S2 is continued on the next page.

Table S2 - Microinversions identified by this study, continued.

INV #	Locus	Intron	Length (bp)	Inverted sequence found in:	Identified bl2seq	Identified "by eye"	Notes
CLTCL1 – (total of 5 sites with inversions)							
19	*CLTCL1*	7	20	*Upupa*	yes	no	
20	*CLTCL1*	7	28	*Chalcopsitta*	yes	no	
21	*CLTCL1*	7	22	Otididae (*Choriotis*, *Eupodotis*)	yes	no	Overlaps with INV-22.
22	*CLTCL1*	7	16	*Crypturellus*	yes	no	Overlaps with INV-21.
23	*CLTCL1*	7	16-22	Palaeognathae or Neognathae	yes	no	Derived form of inversion unclear.
EEF2 – (total of 6 sites with inversions)							
24	*EEF2*	5	18	*Syrrhaptes*	yes	no	
25	*EEF2*	6	21	*Urocolius*	yes	no	
26	*EEF2*	6	15	*Climacteris*	yes	no	
27	*EEF2*	7	14	*Pandion*	yes	no	
28	*EEF2*	8	17	*Jacana*	yes	no	Overlaps with INV-29.
29	*EEF2*	8	23	*Haematopus*	yes	no	Overlaps with INV-28.
FGB – (total of 4 sites with inversions)							
30	*FGB*	5	16	*Charadrius*, *Haematopus*, *Phegornis*	yes	yes	
31	*FGB*	5	5	Sulae (*Anhinga*, *Morus*, *Phalacrocorax*)	no	yes	
32	*FGB*	6	32	*Coturnix*	yes	yes	
33	*FGB*	7	20	*Gallus*	yes	yes	
GH1 – (total of 3 sites with inversions)							
34	*GH1*	3	9-12	Phasianidae (*Coturnix*, *Gallus*, *Numida*, *Rollulus*)	yes	yes	
35	*GH1*	3	36	*Sapayoa*	yes	no	
36	*GH1*	3	13	*Turnix*	yes	no	

Table S2 is continued on the next page.

Table S2 - Microinversions identified by this study, continued.

INV #	Locus	Intron	Length (bp)	Inverted sequence found in:	Identified bl2seq	Identified "by eye"	Notes
HMGN2 – (total of 4 sites with inversions)							
37	*HMGN2*	2	25	*Eurypyga*, *Rhynochetos*	yes	no	
38	*HMGN2*	4	18	*Struthio*	yes	no	
39	*HMGN2*	4	41	*Mesitornis*	yes	no	
40	*HMGN2*	5	28	*Arenaria*	yes	yes	
IRF2 – (total of 2 sites with inversions)							
41	*IRF2*	2	14	*Chalcopsitta*	yes	no	Overlaps with INV-42.
42	*IRF2*	2	28	*Upupa*	yes	no	Overlaps with INV-41.
PCBD1 – (total of 5 sites with inversions)							
43	*PCBD1*	2	14	*Tinamus*	yes	no	
44	*PCBD1*	3	38	*Rhea*	yes	no	
45	*PCBD1*	3	24	*Chauna*	yes	no	
46	*PCBD1*	3	18	*Phegornis*	yes	no	
47	*PCBD1*	3	25	*Tockus*	yes	no	
TGFB2 – (single site with an inversion)							
48	*TGFB2*	5	10	*Colius*	yes	yes	
TPM1 – (single site with an inversion)							
49	*TPM1*	6	24	*Trogon*	yes	yes	
